# Comparison of D&C and hysteroscopy after UAE in the treatment of cesarean scar pregnancy: A case-control study

**DOI:** 10.1097/MD.0000000000028607

**Published:** 2022-01-21

**Authors:** Lili Cao, Zhida Qian, Lili Huang

**Affiliations:** Women's Hospital, Zhejiang University School of Medicine, People's Republic of China.

**Keywords:** cesarean scar pregnancy, curettage, hysteroscopy, uterine artery embolization

## Abstract

Cesarean scar pregnancy (CSP) stands for the severe complication secondary to cesarean section, and its incidence shows an increasing trend recently. However, no consensus has been reached about the CSP treatment. This study aims to explore the necessity of hysteroscopy (H/S) after preventive uterine artery embolization (UAE).

A case-control report. The childbearing CSP patients with a cesarean section history were evaluated by ultrasonography, with a gestational age of less than 10 weeks. Thirty-four patients receiving dilation and curettage (D&C) after UAE were enrolled into the D&C group, whereas 46 undergoing H/S and curettage after UAE were enrolled into the H/S group.

Differences in success rate and decrease in the β-hCG level in serum on the second day of surgery were not significantly different between D&C and H/S groups (*P* > .05). Also, differences in side effect rate (except for the anesthesia-related side effects), intraoperative blood loss amount, postoperative bleeding time, and total length of hospital stay were not significant between 2 groups (*P* > .05). Compared with D&C group, H/S group had decreased postoperative length of hospital stay (*P* < .05), increased hospitalization cost (*P* < .05), and significantly elevated time of CSP mass disappearance (*P* < .05). In addition, 8 (18.19%) patients in H/S group developed anesthesia-related side effects.

This study reveals no obvious difference between UAE + D&C and UAE + H/S in terms of the clinical efficacy and safety, except for the time of CSP mass disappearance and anesthesia-related side effects. The hospitalization cost is more expensive for UAE + H/S, but the postoperative length of stay is shorter for UAE + H/S. UAE + H/S is associated with the risk of anesthesia-associated side effects.

## 1 Introduction

Cesarean scar pregnancy (CSP) refers to the pregnancy where the pregnancy sac is implanted at the uterine scar. CSP represents a rare ectopic pregnancy in a special site and a serious long-time complication secondary to cesarean section (CS).^[1]^ Recently, CSP incidence increases significantly worldwide, especially in China, due to the high rate of CS in the past. More and more females are willing to have another baby after CS as the second-child policy is opened, and the number of CSP cases has increased accordingly.^[2]^

At present, there is no well-recognized optimal treatment for CSP at home and abroad. Although studies have reported cases of successful delivery with CSP,^[3]^ it is still considered that the termination of pregnancy after CSP is a better option.^[4]^ Typically, the mainstream treatment is to pretreat uterine artery embolization (UAE) and then perform dilation and curettage (D&C) under ultrasound guidance.^[5]^ However, D&C can not be performed under direct vision, so the implantation site of the pregnancy sac can not be observed directly, which results in a risk of uterine perforation and major bleeding by D&C alone.

In recent years, the removal of CSP pregnancy tissue under hysteroscopy (H/S) combined with curettage surgery (H/S + D&C) has been considered as a safe and effective minimally invasive treatment.^[6]^ Nonetheless, the H/S + D&C treatment is associated with certain limitations. For example, doctors should be familiar with the hysteroscopic equipment and be experienced in operation. Meanwhile, H/S, together with its anesthesia methods, may bring certain complications and cause increased costs, thus potentially increasing the risk and financial burden on patients.

Therefore, to explore the necessity of H/S after preventive UAE, this study compared the surgical efficacy, safety, costs, and hospital stay of the 2 treatment methods.

## 2 Methods

### 2.1 Subjects

This is a case-control study. CSP was diagnosed according to age, gestational age, medical history, and ultrasound examination of patients. All the enrolled patients were of childbearing age who had previously received CS, with or without menopause. No anatomical abnormality was detected in the reproductive system. All patients did not receive sex hormone therapy, radiation therapy, or chemotherapy within 6 months before the operation. The gestational age of patients enrolled was less than 10 weeks. The present work enrolled CSP patients who met the above conditions who underwent UAE at the Women's Hospital of Zhejiang University School of Medicine from January 2017 to December 2019. This institution represents the greatest gynecology and obstetrics medical center in Zhejiang, China. A total of 80 CSP cases were enrolled for retrospective analysis. The Ethics Committee of Women's Hospital of Zhejiang University School of medicine had approved our study protocol (ID:20180018). All patients signed the informed consent to participate in the study.

**Figure 1:**
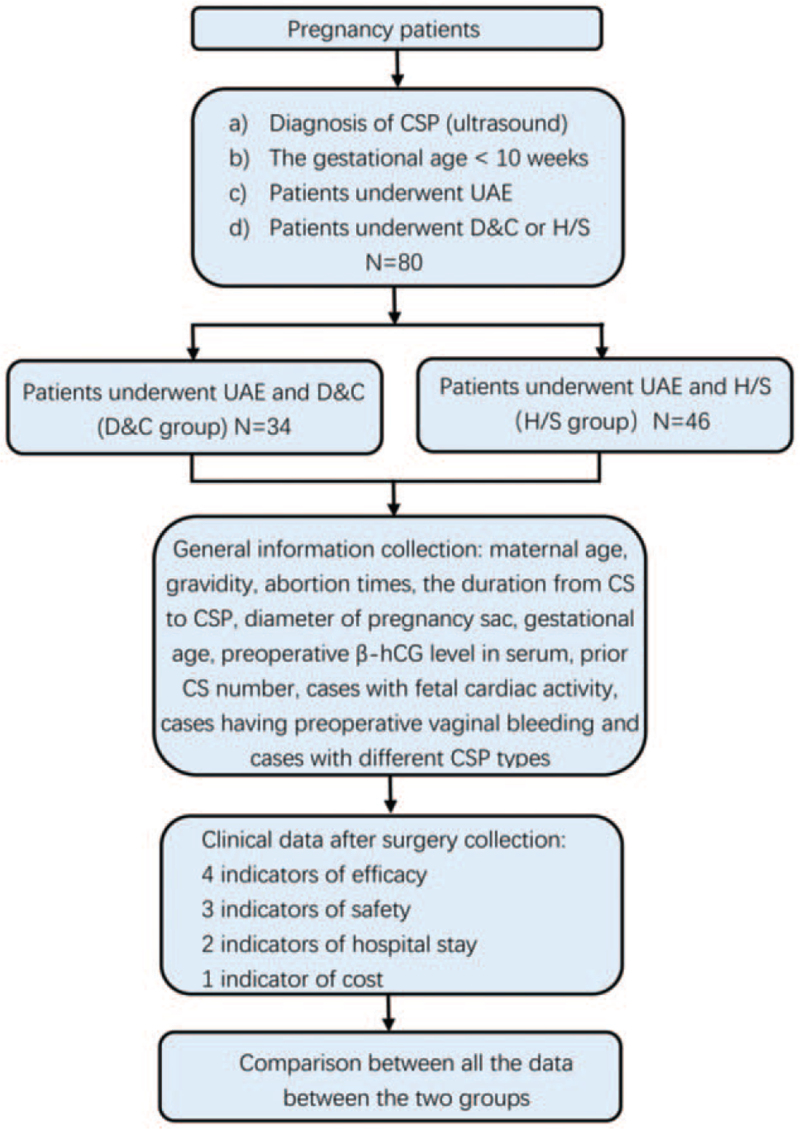
Flow chart of data collection. CS = cesarean section, CSP = caesarean scar pregnancy, D&C = dilation and curettage, H/S = hysteroscopy, UAE = uterine artery embolization.

A comprehensive flow sheet abstract of the data collection was showed as Figure 1.

### 2.2 The diagnosis standard of the gestational type CSP by ultrasonography

The ultrasonic diagnostic criteria for CSP patients are as follows^[7]^: pregnancy sac is not detected within cervical canal or uterine cavity; pregnancy sac is seen at scar site on the lower uterus anterior wall, with thinning and interrupted muscle layer; color Doppler flow imaging shows high velocity flow signals with low impedance surrounding pregnancy sac, and resistance index (RI) is generally <0.4 to 0.5.

Generally, CSP can be classified as 2 types,^[8]^ namely, gestational sac type and asymmetrical mass type. As for gestational sac type, the gestational sac is embedded into the muscle layer and grows to the bladder or attaches to the scar and grows to the uterine cavity. At the attachment of pregnancy sac, the myometrium may be absent or thin. The asymmetrical mass type is rarer than the former, in which the lesion is mainly mass echo that is solid or cystic in the lower part of the anterior wall. All the patients in this study were with gestational sac type.

**Figure 2:**
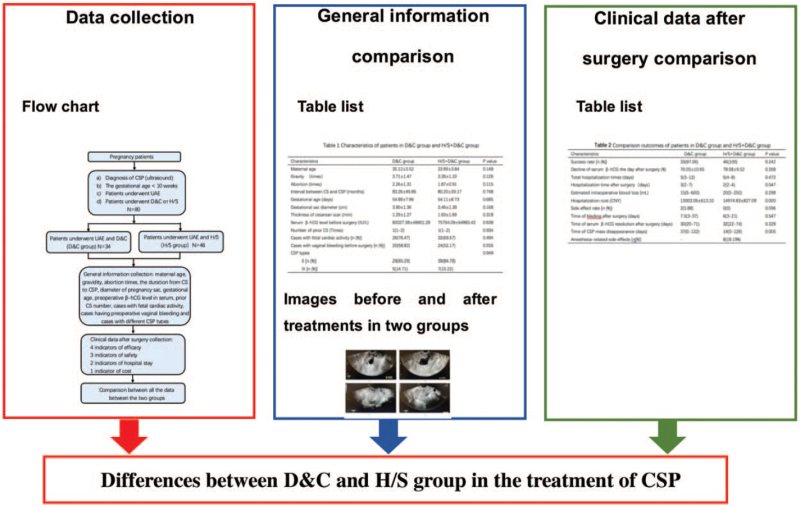
The infographic abstracts of the article. CS = cesarean section, CSP = caesarean scar pregnancy, D&C = dilation and curettage, H/S = hysteroscopy, UAE = uterine artery embolization.

Also, CSP can be further divided into 3 types according to the pregnancy sac growth direction and myometrial thickness between the bladder and the fetal sac.^[9]^ In type I, partial or most gestational sac is located in uterine cavity, with thinning myometrium lying between bladder and pregnancy sac, and a thickness of >3 mm. If the thickness is ≤3 mm, the CSP is defined as type II. In type III, the pregnancy sac is convex toward the bladder, with significantly thinning or missing myometrium between bladder and pregnancy sac and a thickness of ≤3 mm. Usually, UAE is adopted for type II and type III CSP in this hospital (Fig. 2).

### 2.3 Management

#### 2.3.1 UAE pretreatment

All patients received preventive UAE at 24 hour before D&C or H/S. Specifically, the UAE pretreatment was performed by 2 experienced radiologists under local anesthesia by adopting the super selective “Seldinger” technology, and the bilateral uterine arteries were embolized with gelatin sponge particles. Embolization was confirmed by post-embolization angiography.

#### 2.3.2 Dilation and curettage

**Figure 3:**
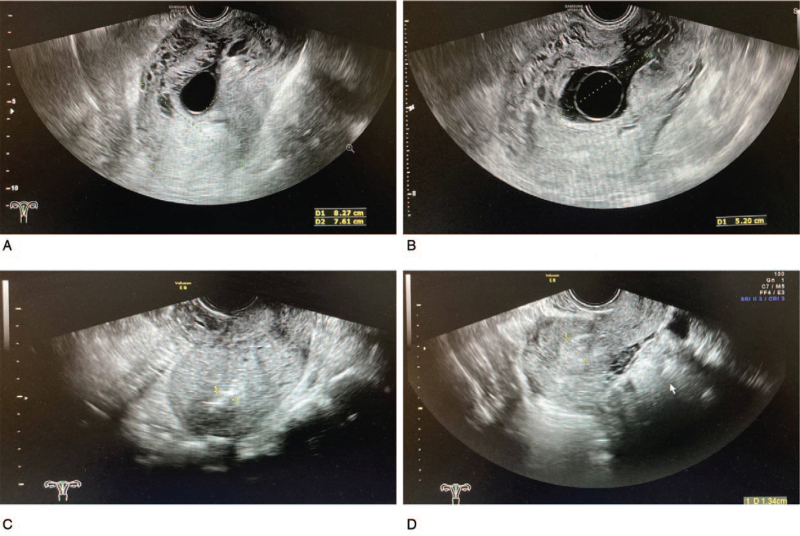
CSP ultrasonography. A. Transvaginal ultrasonography revealing CSP before D&C. B. Transvaginal ultrasonography showing CSP before H/S. C. Transvaginal ultrasonography showing CSP after D&C. D. Transvaginal ultrasonography showing CSP after H/S. CSP = caesarean scar pregnancy, D&C = dilation and curettage, H/S = hysteroscopy.

Thirty-four patients receiving D&C were enrolled into the D&C group. All D&C surgeries were operated by an experienced gynecologist under the assistance of transabdominal ultrasound. First of all, the cervix was carefully expanded. Then, electric negative pressure suction and local curette scraping were applied for removing pregnancy sac along with the local hemorrhage. For reducing risks of major bleeding and uterine perforation, residual pregnancy tissue was removed through scraping gently. Uterine ultrasonography before and after surgery is shown in Figure 3A and 3C, respectively.

#### 2.3.3 H/S and curettage

Forty-six patients undergoing H/S and D&C were enrolled into the H/S group. All H/S and D&C surgeries were operated by an experienced gynecologist. H/S was conducted to check the implantation site of pregnancy sac, together with uterine cavity condition. Then, under the transabdominal ultrasound surveillance, the oval forceps were used to force out the pregnancy sac and its attachment. Local electrocoagulation was applied in treating bleeding points. After satisfactory inspection, this operation was completed. Uterine ultrasonography before and after surgery is presented in Figure 3B and 3D, respectively.

### 2.4 Research contents

For the 2 groups of patients, their clinical features before surgery were extracted, including maternal age, gravidity time, abortion time, the time duration from CS to CSP, diameter of pregnancy sac under ultrasound, gestational age, thickness of cesarean scar, preoperative β-hCG level in serum, prior CS number, fetal cardiac activity, vaginal bleeding, and CSP types.

Clinical data were collected from these 2 groups of patients after surgery, including 4 indicators of efficacy (the declined blood β-HCG level on the first day following operation, bleeding time, β-hCG concentration in serum and mass disappearance time of CSP after surgery), 3 indicators of safety (successful rate of surgery, intraoperative blood loss amount and the surgery-related complications), 1 indicator of cost (hospitalization cost) and 2 indicators of hospital stay (total length of hospital stay and postoperative length of stay). In this study, the complications were hemorrhagic shock, infection and anesthesia-related side effects. All patients were followed up after surgery. The blood β-HCG level was reviewed once a week until it returned to physiological level (<5.3 IU/L). In addition, transvaginal ultrasound was reviewed at 1, 2, and 3 months postoperatively to observe the local residues in uterine until they completely disappeared.

### 2.5 Statistical methods

SPSS 20.0 (IBM, the USA) was employed for data analysis. *P* value indicated the 2-sided probability and a difference of *P* < .05 indicated statistical significance. First of all, all patients were subjected to the Kolmogorov-Smirnov test (normal distribution test) together with the variance homogeneity test (Levene Test). For normally distributed data with homogenous variance, the independent sample *t* test was used, whereas for abnormally distributed data with heterogeneous variance, the Mann–Whitney *U* Test (non-parametric test) was adopted. Meanwhile, data regarding the composition ratio were measured by the chi-square test (*χ*^2^ test).

## 3 Results

### 3.1 General information

There were no statistical differences in maternal age, gravidity, abortion times, the duration from CS to CSP, diameter of pregnancy sac, gestational age, preoperative β-hCG level in serum, prior CS number, cases with fetal cardiac activity, cases having preoperative vaginal bleeding or cases with different CSP types between D&C and H/S groups (*P* > .05, Table 1).

**Table 1:** Characteristics of patients in D&C group and H/S + D&C group.

Characteristics	D&C group	H/S + D&C group	*P* value
Maternal age	35.12 ± 3.52	33.89 ± 3.84	.148
Gravity (times)	3.71 ± 1.47	3.26 ± 1.10	.126
Abortion (times)	2.26 ± 1.31	1.87 ± 0.91	.115
Interval between CS and CSP (mo)	83.26 ± 45.86	80.20 ± 39.17	.748
Gestational age (d)	54.88 ± 7.96	54.11 ± 8.73	.685
Gestational sac diameter (cm)	3.90 ± 1.36	3.46 ± 1.39	.168
Thickness of cesarean scar (mm)	1.29 ± 1.27	1.63 ± 1.69	.318
Serum β-hCG level before surgery (IU/L)	82027.38 ± 48901.28	75754.09 ± 64865.42	.638
Number of prior CS (times)	1 (1-2)	1 (1-2)	.934
Cases with fetal cardiac activity [n (%)]	26 (76.47)	32 (69.57)	.494
Cases with vaginal bleeding before surgery [n (%)]	20 (58.82)	24 (52.17)	.555
CSP types			.949
II [n (%)]	29 (85.29)	39 (84.78)	
III [n (%)]	5 (14.71)	7 (15.22)	

### 3.2 Comparison of efficacy indicators

The clinical outcome data are displayed in Table 2. Clearly, there were no statistically significant differences in the reduced β-hCG level in serum on the second day following operation (76.05 ± 10.65 vs 78.58 ± 9.52%, *P* = .268), bleeding time [7.5(3-37) vs 6(3-21), *P* = .547] or serum β-hCG resolution [30(20-71) vs 32(22-74), *P* = .329] between D&C and H/S groups.

**Table 2:** Comparison outcomes of patients in D&C group and H/S + D&C group.

Characteristics	D&C group	H/S + D&C group	*P* value
Success rate [n (%)]	33 (97.06)	46 (100)	.242
Decline of serum β-hCG the day after surgery (%)	76.05 ± 10.65	78.58 ± 9.52	.268
Total hospitalization times (d)	5 (3-12)	5 (4-8)	.472
Hospitalization time after surgery (d)	3 (2-7)	2 (2-4)	.047
Estimated intraoperative blood loss [mL)	15 (5-600)	20 (5-250)	.298
Hospitalization cost (CNY)	13002.06 ± 613.10	14974.83 ± 827.09	.000
Side effect rate [n (%)]	2 (5.88)	0 (0)	.096
Time of bleeding after surgery (d)	7.5 (3-37)	6 (3-21)	.547
Time of serum β-hCG resolution after surgery (d)	30 (20-71)	32 (22-74)	.329
Time of CSP mass disappearance (d)	37 (0-132)	14 (0-126)	.005
Anesthesia-related side effects [n (%)]	-	8 (18.19%)	

Compared with D&C group, the time of CSP mass disappearance was significantly shorter in H/S group [37(0-132) vs 14(0-126) days, *P* = .005].

### 3.3 Comparison of safety indicators

Difference in success rate was not statistically significant between D&C and H/S groups (97.06% vs 100%, *P* = .242). Among the 80 patients enrolled, 79 patients were treated successfully, whereas 1 in D&C group failed in the treatment. This patient developed vaginal bleeding with infection for 23 days after D&C surgery. On the 23rd day after D&C, the patient suffered from hemorrhagic shock due to massive vaginal bleeding, so she was admitted again for D&C surgery with UAE pretreatment. Unfortunately, it failed again. H/S was performed due to the poor surgical results of D&C. Eventually, the blood loss amount in this patient was significantly reduced, and villous tissue was detected in the blood clot removed from the scar.

Differences in incidence of operation-related side effects [2(5.88) vs 0(0), *P* = .096], and intraoperative blood loss amount [15(5-600) vs 20(5-250), *P* = .298] between D&C and H/S groups were not statistically significant.

Difference in incidence of side effects showed no statistical significance between D&C and H/S groups [2(5.88%) vs 0(0), *P* = .096]. Two patients in D&C group developed a pelvic infection, of them, 1 was the failed case mentioned above, while the other 1 was discovered with pelvic infection at 7 days after surgery. The patient suffered from vaginal bleeding at 17 days preoperatively and was discharged at 3 days after surgery. All patients in H/S group had no serious side effects (pelvic infection or diffuse intravascular coagulation). During the H/S surgery, 15 patients had intrauterine adhesions and were treated with operative H/S.

Eight patients in H/S group suffered from the anesthesia-related side effects. Among them, 1 had headache, and no treatment was performed except for emotional comfort. The headache in the patient disappeared 1 day after the surgery. In addition, 7 of these patients had urinary retention and all of them recovered after catheterization. Besides, 1 of the 7 patients with urinary retention was positive for occult blood due to catheter friction.

### 3.4 Comparison of cost indicator

Compared with D&C group, H/S group had remarkably increased hospitalization cost (13002.06 ± 613.10 vs 14974.83 ± 827.09 CNY, *P* = .000).

### 3.5 Comparison of hospital stay indicators

Compared with D&C group, H/S group had significantly decreased postoperative length of stay [3(2-7) vs 2(2-4) days, *P* = .047].

Difference in total length of hospital stay was not statistically significant between D&C and H/S groups [5(3-12) vs 5(4-8), *P* = .472].

## 4 Discussion

This study found that, differences in the success rate and decreased β-hCG level in serum on the first day following operation were not significant between D&C and H/S groups. CSP, as a special type of ectopic pregnancy, has a blood β-hCG level similar to the normal level of intrauterine pregnancy. No matter which treatment method is adopted, it may experience residual trophoblasts. Therefore, regularly monitoring the changes in serum β-hCG level after surgery is of great significance to judge the effect of surgical treatment. In the present work, postoperative β-hCG resolution time in serum of D&C and H/S groups was 30 (20-71) and 32 (22-74), respectively, which seemed to be longer than that reported in previous studies.^[10]^ For all the studies mentioned in that article, the serum β-hCG resolution time was less than 1 month. One possible reason for this phenomenon is that different surgical methods were used. To prevent uterine rupture, doctors just remove the pregnancy tissue as much as possible, which may not be the same as transvaginal hysterotomy.

Also, differences in the incidence of operation-related side effects (pelvic infection and diffuse intravascular coagulation), intraoperative blood loss amount, postoperative bleeding time, and total length of hospital stay were not significant. However, compared with D&C group, H/S group had decreased postoperative length of hospital stay, increased hospitalization cost, and significantly decreased time of CSP mass disappearance. The length of hospital stay and the hospitalization cost will partially affect the patient's choice of treatment. In China, the price of hysteroscopic surgery is more expensive than that of D&C. The reason why the average postoperative hospital stay after D&C is longer than that of H/S treatment may be for the blindness of D&C, which prolongs the observation time of patients in the hospital after surgery. Another study also found that H/S surgery in treatment of small mass endogenous CSP is effective, shorter hospitalization time and quick recovery.^[11]^ Although the time of CSP mass disappearance was different in the 2 groups, their time of serum β-hCG resolution was similar, indicating a high possibility that the existing mass was the parent tissue. Qiu et al^[12]^ also have the similar results for the comparison between patients with the 2 treatments.

One patient in D&C group failed. This may be related to the blindness of D&C surgery, which is performed with indirect vision and villi tissue could not be cleaned up in time. Fifteen patients in H/S group were found to develop intrauterine adhesions, and they were treated with operative H/S. For patients with the demands for pregnancy, it can help them avoid another operation for intrauterine adhesions.

This study indicated that, anesthesia brought additional side effects to the patients. Generally, urinary retention is the most likely side effect of anesthesia, which can be completely relieved after catheterization. However, the insertion of a urinary catheter may also cause damage to the ureter. These anesthesia-related side effects are not serious, but they may affect the patient's choice of surgical method. Some patients who lack the anesthesia knowledge may not know which anesthesia method to choose. In addition, patients have a deep fear of the possible anesthesia-associated side effects, in this regard, they may prefer the D&C surgery. Also, patients may have no idea of symptom severity and are worried about the use of anesthesia. In this case, the surgeon and anesthetist should communicate with the patients before the operation, so as to ease their nervousness. Noteworthily, the precise use of anesthetic drugs can effectively reduce the risk of anesthesia-related side effects and the doctors can well manage the possible complications.

Limitation of the study: Firstly, in this study, all patients were pretreated with UAE. Due to the lack of control, it remains to be further investigated about whether UAE plays an important role, and whether it is sufficient to affect the use of H/S. So far, there have been many methods used to treat CSP, treatments like MTX injection, UAE alone are not recommended for treatment of CSP due to their low successful rate and high complication rates.^[13]^ UAE combined with D&C or H/S are reported with low complication rates.^[14,15]^ Secondly, there were only less than 10 cases with mass CSP from January, 2017 to December, 2019, and most of them had received H/S previously; as a result, it was not analyzed in this study.

In conclusion, both D&C and H/S after UAE can successfully terminate the gestational type CSP. Apparently, H/S displays significant advantages in terms of the time of CSP mass disappearance and the postoperative length of stay. Nonetheless, the application of H/S results in higher hospitalization cost and brings additional anesthesia-related risks. Therefore, treatment should be individualized based on the patient characteristics, meanwhile, the patient feelings should be considered, and their wishes should be fully respected. Also, the medical conditions of each hospital and the technical skills of doctors are different, which should be considered when selecting an appropriate surgical method. According to this study, UAE followed by D&C is more suitable for patients with the gestational age of less than 70 days who are worried about anesthesia and high cost. H/S is more appropriate for patients who are highly suspected of intrauterine adhesions before surgery and are willing to get pregnant again.

Future direction: In the future, we will further implement a cohort study to verify the differences between D&C and H/S after UAE. And we will find out the risk factors that lead to the failure of different treatment methods, to provide a more reliable basis for the treatment of CSP patients.

## Author contributions

Lili Cao has made contributions to the acquisition, analysis, and interpretation of data. Zhida Qian has made contributions to the design of the work. Lili Huang has made contributions to the conception.

**Conceptualization:** Lili Huang.

**Data curation:** Lili Cao.

**Formal analysis:** Lili Cao.

**Funding acquisition:** Zhida Qian.

**Supervision:** Zhida Qian.

**Writing – original draft:** Lili Cao.

**Writing – review & editing:** Zhida Qian.
